# Radiomic and Clinical Model in the Prognostic Evaluation of Adenoid Cystic Carcinoma of the Head and Neck

**DOI:** 10.3390/cancers16233926

**Published:** 2024-11-23

**Authors:** Paolo Rondi, Michele Tomasoni, Bruno Cunha, Vittorio Rampinelli, Paolo Bossi, Andrea Guerini, Davide Lombardi, Andrea Borghesi, Stefano Maria Magrini, Michela Buglione, Davide Mattavelli, Cesare Piazza, Marika Vezzoli, Davide Farina, Marco Ravanelli

**Affiliations:** 1 Radiology Unit, Department of Medical and Surgical Specialties, Radiological Sciences and Public Health, University of Brescia, Spedali Civili, Piazzale Spedali Civili 1, 25123 Brescia, Italy; 2 Otolaryngology Unit, Department of Medical and Surgical Specialties, Radiological Sciences and Public Health, University of Brescia, Spedali Civili, Piazzale Spedali Civili 1, 25123 Brescia, Italy; 3 Neuroradiology Department, Unidade Local Saúde Braga, 4710-243 Braga, Portugal; 4 Department of Biomedical Sciences, Humanitas University, Via Rita Levi Montalcini 4, Pieve Emanuele, 20072 Milan, Italy; 5 Humanitas Cancer Center, IRCCS Humanitas Research Hospital, Via Manzoni 56, Rozzano, 20089 Milan, Italy; 6 Department of Radiation Oncology, Istituto del Radio O. Alberti, University of Brescia and Spedali Civili Hospital, 25123 Brescia, Italy; 7 Department of Molecular and Translational Medicine, Unit of Biostatistics, University of Brescia, 25133 Brescia, Italy

**Keywords:** radiomics, adenoid cystic carcinoma, salivary gland neoplasms, data augmentation, relapse-free survival

## Abstract

Background/Objectives: Adenoid Cystic Carcinoma (AdCC) is a rare malignant salivary gland tumor, with high rates of recurrence and distant metastasis. This study aims to stratify patients Relapse-Free Survival (RFS) using a combined model of clinical and radiomic features from preoperative MRI. Methods: This retrospective study included patients with primary AdCC who underwent surgery and adjuvant radiotherapy. Segmentations were manually performed by two head and neck radiologists. Radiomic features were extracted using the 3D Slicer software. Descriptive statistics was performed. A Survival Random Forest model was employed to select which radiological feature predict RFS. Cox proportional hazards models were constructed using clinical, radiological variables or both. Synthetic data augmentation was applied to address the small sample size and improve model robustness. Models were validated on real data and compared using the C-index and Prediction Error Curves (PEC). Results: Three Cox models were developed: one with clinical features (C-index = 0.67), one with radiomic features (C-index = 0.68), and one combining both (C-index = 0.77). The combined clinical-radiomic model had the highest predictive accuracy and outperformed models based on clinical or radiomic features. The combined model also exhibited the lowest mean Brier score in PEC analysis, indicating better predictive performance. Conclusions: This study demonstrate that a combined radiomic-clinical model can predict RFS in AdCC patients. This model may provide clinicians a valuable tool in patient’s management and may aid in personalized treatment planning.

## Simple Summary

Adenoid Cystic Carcinoma (AdCC) is a rare malignancy of the head and neck and is a heterogenous tumor both histologically and clinically; its prolonged asymptomatic clinical course often leads to a delayed diagnosis. Moreover, the risk of local and distant recurrence also years after primary treatment is high. In this setting, the ability to stratify patients’ prognosis is crucial to better identify patients at risk of recurrence. This article explores a radiomics and clinical model to evaluate the Relapse-Free Survival (RFS) in patients with AdCC.

## 1. Introduction

Adenoid Cystic Carcinoma (AdCC) is a rare epithelial malignant tumor most commonly arising from the salivary glands. AdCC is slightly more common in females, with a median age at diagnosis in the 6th decade [1]. Long-term prognosis is generally poor, with 10-year survival estimates mostly ranging from 52% to 67% [2,3,4].

Patients with AdCC often present with loco-regional and distant recurrences, with a highly variable disease-free interval of up to 10 years after first diagnosis [4,5]. Although new approaches are under investigation [6], therapeutic options for recurrent/metastatic AdCC are limited, with current protocols of systemic treatment showing unsatisfactory response rates [7]; for this reason, primary treatment radicality is of paramount importance [8].

The standard of care is surgery followed by adjuvant radiotherapy [7]; the preoperative radiological assessment of salivary gland malignancy shows a largely varying accuracy, depending on the location of tumors and the expertise of radiologists [9,10].

Since in major salivary glands, the preoperative diagnosis is usually accomplished through a cytological evaluation, the radiological examination is of value in contributing to differential diagnosis and tumor staging. More recently, the application of radiomics to salivary gland oncology has offered valuable input for the development of artificial-intelligence-assisted tools for differential diagnosis and prognosis stratification [11,12,13,14].

Data augmentation consists of the creation of synthetic patients and data using a complex deep-learning algorithm, and it is particularly useful in research on rare diseases. Obtaining a large enough sample size for meaningful statistical analysis can be challenging. As more researchers use synthetic data, its credibility and acceptance in the scientific community continue to grow.

Although in the literature several studies have highlighted the value of radiomics in differentiating benign from malignant salivary gland tumors [15,16] and distinguishing different histotypes [17,18], no studies have been published on applying radiomics specifically for AdCC.

The current study aims to stratify patients affected by primary AdCC of the salivary glands and treated with surgery with curative intent based on texture analysis of preoperative MRI. The development of a predictive model based on a combined clinical-radiomic model can move beyond a “one-size-fits-all” approach to a more tailored patient care: the use of radiomics could assist in the earlier identification of patients at risk of metastasis, enabling earlier intervention strategies.

## 2. Materials and Methods

This retrospective observational study was performed in line with the principles of the Declaration of Helsinki. Ethical approval was obtained from the local ethical committee (NP4266).

### 2.1. Patients

All patient data were anonymized to ensure confidentiality and comply with ethical standards.

A retrospective analysis of patients affected by primary salivary malignancies from 1995 to 2020 was conducted at the Department of Otolaryngology—Head and Neck Surgery, University of Brescia, Italy.

Inclusion criteria were: (a) diagnosis of treatment-naïve primary salivary gland AdCC, (b) treated with upfront surgery with curative intent and adjuvant radiotherapy, (c) availability of preoperative Magnetic Resonance Imaging (MRI) within one month prior to surgery (including turbo-spin-echo (TSE) T2-weighted sequences). Exclusion criteria were: (a) unavailability of adequate follow-up (at least 6 months after treatment), (b) distant metastasis at diagnosis, and (c) inadequate image quality due to artifacts.

Regarding follow-up, clinical examination and MRI were performed every 4 months for the first two years and every 6 months after that. A chest CT scan was performed annually.

### 2.2. Image Analysis

Magnetic Resonance (MR) images in Digital Imaging and Communications in Medicine (DICOM) format were transferred from the institutional Picture Archiving and Communication System (PACS) to an offline workstation equipped with the open-source software 3D Slicer version 5.5.0 (www.slicer.org, accessed on 20 October 2024) [19].

Tumors were segmented manually separately by two head and neck radiologists with 5 years of experience. Tumor segmentation was conducted on TSE T2-weighted images enclosing the whole tumor volume.

The following preliminary steps were performed before feature extraction: correction for magnetic field inhomogeneity by means of N4-ITK filter, voxel size resampling to 1 × 1 × 1 mm^3^, histogram discretization with a fixed bin width of 25. Radiomic features were extracted using the 3D Slicer extension of pyradiomics and included the following semantic groups: first-order statistics and texture features. The features were extracted from original images and after a Laplacian of Gaussian (LoG) filtering using sigma corresponding to 2 and 4 mm. Overall, 279 radiomic features were extracted for each patient.

### 2.3. Statistical Analysis

Medical records were retrieved to collect baseline demographics and clinical, tumor-, and treatment-related characteristics. Descriptive statistics on quantitative variables included median, interquartile range (IQR) and min-max; for qualitative variables, absolute and percentage frequencies were computed. Moreover, radiological features were collected, and the Interclass Correlation Coefficient (ICC) was used to evaluate the consistency between results obtained from two different readers. Only features with an ICC > 0.9 were included in the final dataset.

Using the Kaplan–Meier survival curves, the Relapse-Free Survival (RFS) probabilities were estimated at specific time points (up to 1 year, 3 years, and 5 years post-treatment). The data were censored at these intervals to ensure an accurate representation of survival probabilities.

For identifying which of the clinical variables impact the outcome, namely the RFS, univariate Cox analysis [20] was computed on the dataset, and variables with *p*-value < 0.05 were selected.

Since radiomics features, jointly with clinical variables, could increase the predictive accuracy of a survival model, a variable selection approach was used to identify those variables that have a strong impact on the RFS. In doing so, the Relative Variable Importance Measure (relVIM) extracted from the Survival Random Forest [21,22] (SFR, which is a multivariate model) was used as a variable selection method for detecting which radiological features (covariates) predict the RFS (outcome). This procedure was not applied to the clinical data due to the presence of missing values across variables. In fact, the Survival Random Forest method requires the imputation of missing data for model estimation, but, in correspondence with the small sample size, there are significant concerns that such imputation could result in biased and unreliable outcomes.

The primary concern with multivariate analysis is the small sample size, a common situation in the presence of rare diseases, which prevents splitting the observations into training and test sets. To extend the sample size, it is possible to use procedures that increase observations of a real data set. They create artificial datasets that mimic the statistical characteristics of real data and help to validate findings from small patient cohorts, ensuring that conclusions drawn are not due to chance. In particular, the synthpop package [23] in R is a tool able to generate synthetic versions of real datasets. It builds on methodologies akin to multiple imputations and adapts them for the purpose of data synthesis. It replaces original data values with sampled values from estimated probability distributions. Data synthesis is developed using CART (Classification and Regression Trees), a non-parametric approach. However, for testing and validation purposes, it is crucial to use real data only. This approach ensures that the performance metrics obtained when estimating a model on synthetic data reflect the model’s true capability to handle new, real-world data, and this keeps the evaluation honest and relevant.

After reducing the number of covariates, considering only clinical and radiological variables that impact RFS prediction, the data were doubled using the synthpop package. With a limited number of covariates, convergency problems are solved, and a classical survival model was estimated using synthetic data for the training phase and real data to test the validity of the predictions obtained. The predicted risk score was derived from the Cox proportional hazards model. To identify the optimal cut-point that maximizes the difference in survival outcomes between two groups, the model evaluates several potential cut-points selecting the one that results in the most significant log-rank test statistic, indicating the greatest separation between the survival curves of the two subgroups of patients.

Three Cox proportional hazards models were estimated to predict RFS (outcome) using distinct sets of covariates: (1) clinical variables identified through univariate Cox analysis on the original dataset; (2) radiomic features selected by the Survival Random Forest (SRF) approach; and (3) a combined model incorporating both clinical and radiomic variables. For each model, the Proportional Hazards (PH) assumption was controlled using Schoenfeld residuals for individual covariates and for the global model. Finally, the performances of these different models were compared by means of the C-Index (which measures the ability of the model to correctly rank the survival times based on the predicted risk scores) and visualized by means of the Prediction Error Curves [24] (PEC, which computes the aggregate Brier score). These procedures help to select the best model among the three estimated.

## 3. Results

Three hundred and eighty consecutive patients affected by primary salivary malignancies were retrieved. Among them, 104 were Adenoid Cystic Carcinoma. Fifty-two patients met the selection criteria. Table 1 resumes baseline patient and tumor characteristics.

Patients were mostly female (67.3%), with a median age of 53 years (IQR: 38–61). The majority of AdCC originated in the minor salivary glands (79.6%), with the sinonasal tract as the most frequent subsite of origin (44.9%). High-grade AdCCs were 32.7% [25,26].

Locally advanced lesions (pT4) were common (75.5%), mostly due to infiltration of adjacent named nerves, muscles, and bones. Nodal metastases were less common (18.4%), although frequently presenting extra-nodal extension (14.3%).

Perineural invasion was almost always described (91.1%), whereas lymphovascular invasion was less frequent (24.4%). As preventable, wide negative margin resections (R0) were not common (13, 28.8%). Only in two cases (4.1%) adjuvant RT was not delivered.

Median follow-up (calculated with the method of reverse Kaplan–Meier) was 112 months (IQR: 92–140). At the end of the current study, 20 patients were deceased, with a median time of survival of 47 months (IQR: 12–95.2), whereas disease relapse was diagnosed in 30 patients, with a median time of recurrence-free survival of 25.5 months (IQR: 3.7–54.5).

An extended table with a univariate Cox analysis of all the variables considered in this study for identifying the clinical variables associated with Relapse-Free Survival (RFS) may be found in the Supplementary Material Table S1.

RFS probabilities estimated at 1-, 3-, and 5-years post-treatment were equal to 0.73, 0.63, 0.47, respectively.

To identify which clinical variables are related to the outcome, univariate Cox analysis was performed on the original dataset (results shown in Table S1). In this analysis, RFS was the outcome, and 51 models were estimated, each using one clinical variable as a covariate at a time. This was the unique applicable strategy due to the high number of missing values in the clinical data. Only grading and margins are significantly associated with RFS (*p*-value < 0.05).

**Figure 1:**
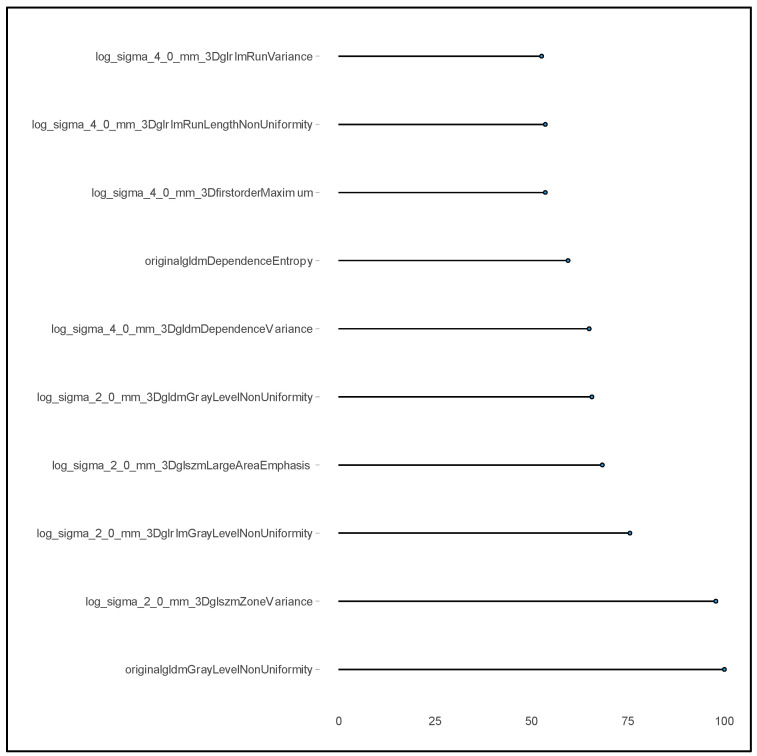
Relative Variable Importance (relVIM), extracted from a Survival Random Forest, where the response variable was Relapse-Free Survival and the 233 radiomic features were the covariates.

In the case of radiomics features that do not contain missing values, to identify those variables that have a strong impact on the prediction of the outcome (i) an SRF multivariate model was estimated where the response variable was RFS and the 233 radiomic features included the covariates, and (ii) the relVIM was extracted from it. Out of the 233 radiomic features which resulted repeatable (ICC > 0.9), only 10 were selected based on a Relative Variable Importance Measure (Figure 1 reports important radiomics features with a relVIM > 50).

Having selected a limited number of clinical and radiological variables by means of two different procedures, it is now possible to estimate a classic survival model. Indeed, convergence issues are no longer a concern, as the data matrix is no more ill-conditioned.

Before proceeding with the estimation of the model, it was decided to employ data augmentation techniques to double the number of observations in the dataset. This approach allowed for the division of the dataset into training and test sets to validate the model estimated on a portion of fresh data. Consequently, as mentioned before, the survival model was trained on synthetic data (52 observations) and then tested on real data (the remaining 52 observations). The synthetic data obtained mimics the existing structure in the original data very well, effectively preserving the key characteristics and relationships existing in them (data shown upon request). This ability to faithfully reproduce the original structure ensures that synthetic data can be effectively used in the training phase of a model without compromising the integrity of the original information.

Three different Cox models were estimated on the training sample containing synthetic data, where the outcome was RFS and the covariates were: (I) the two clinical variables selected by the univariate analysis (grading and margins), (II) the ten radiomic features selected by the SRF (their names are reported in Figure 1), and (III) grading and margin plus the ten radiomics features reported in Figure 1.

**Figure 2:**
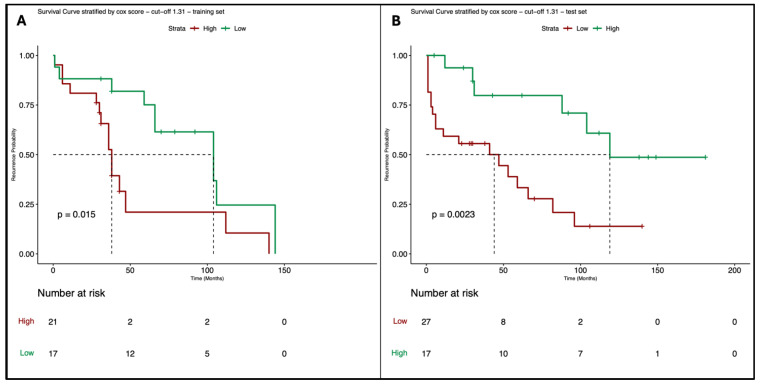
Survival curves of the Cox model where the outcome is RFS and the clinical covariates are grading and margin, stratified respect the best cut-off point estimated on the training set with the long-rank test; (**A**): survival curves estimated on the training set (52 synthetic data); (**B**): survival curves validated on the test set (52 real observations).

In the first Cox model estimated using grading and margin as covariates for predicting the Relapse-Free Survival (outcome), the optimal cut-off point identified with the long-rank test is 1.31, and the corresponding survival curves, which are significantly different (*p*-value = 0.015), are reported in Figure 2A. The Schoenfeld residuals test confirms that the PH assumption is satisfied for each single covariate (grading *p*-value = 0.78, margin *p*-value = 0.57) and for the overall model (*p*-value = 0.83). The model obtained was then tested on the real data using the same cut-off point identified in training, which provided similar results (Figure 2B, *p*-value = 0.0023).

**Figure 3:**
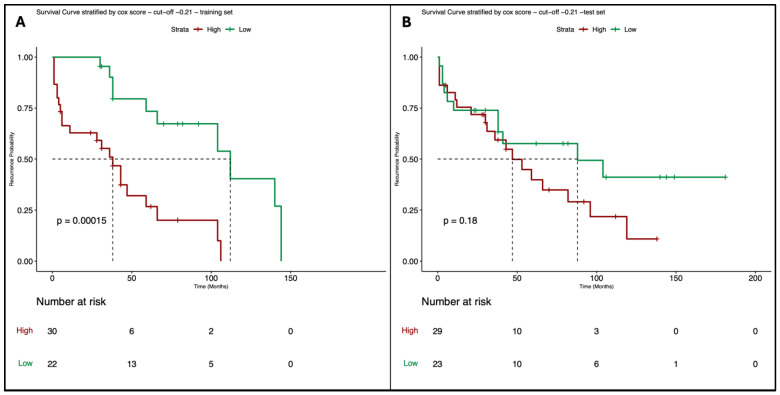
Survival curves of the Cox model where the outcome is RFS and the covariates are the ten radiological features selected by the Survival Random Forest, stratified respect the best cut-off point estimated on the training set with the long-rank test; (**A**): survival curves estimated on the training set (52 synthetic data); (**B**): survival curves validated on the test set (52 real observations).

In the second Cox model estimated using the ten radiological features selected by SRF as covariates for predicting the outcome RFS, the optimal cut-off point is -0.21, and the corresponding survival curves, which are significantly different (*p*-value = 0.00015), are reported in Figure 3A. The Schoenfeld residuals test confirms that the PH assumption is satisfied for each single covariate (all the *p*-values > 0.05) and for the overall model (*p*-value = 0.61). With respect to the first model, the survival curves in the test set do not exhibit a statistically significant difference (Figure 3B, *p*-value = 0.18).

**Figure 4:**
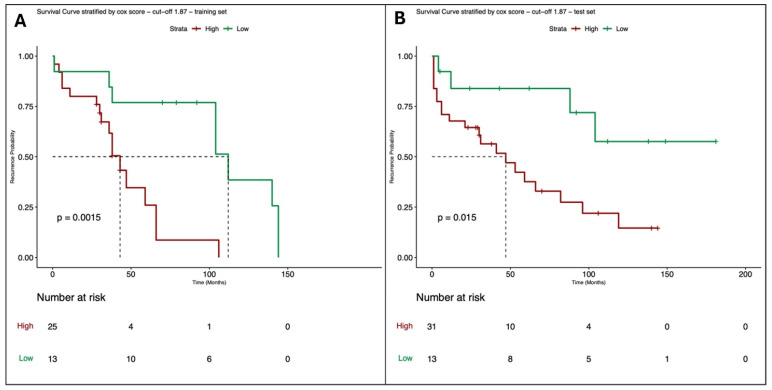
Survival curves of the Cox model where the outcome is RFS and the covariates are the two clinical variables (grading and margin) and the ten radiological features selected by the Survival Random Forest, stratified with respect to the best cut-off point estimated on the training set with the long-rank test; (**A**): survival curves estimated on the training set (52 synthetic data); (**B**): survival curves validated on the test set (52 real observations).

In the third and last Cox model, which incorporates the clinical (grading and margin) and radiological features (ten variables selected by SRF) as covariates for predicting the RFS, the optimal cut-off point is 1.87. Again, the Schoenfeld residuals test confirms that the PH assumption is satisfied for all individual covariates (all *p*-values > 0.05) as well as for the overall model (*p* = 0.51). Corresponding survival curves, which show a significant difference (*p*-value = 0.0015), are reported in Figure 4A. The model obtained was tested on the real data using the same cut-off point identified in training, obtaining similar results to the training set (Figure 4B, *p*-value = 0.015).

Table 2 reports the Cox regression estimates (HR) for the three estimated models, corresponding to 95% CI and *p*-value.

To select the best model among the three estimated, their performances were compared using the C-index (Table 3). The model with the highest C-index is that which is based on a combination of clinical and radiological covariates whose value (greater than 0.7) ensures a good predictive ability.

**Figure 5:**
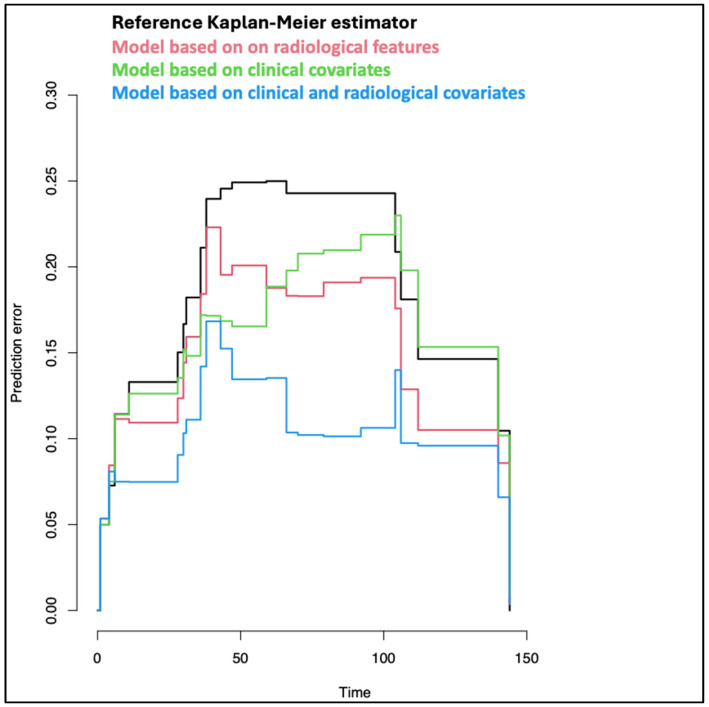
Prediction Errors Curves (PEC) for selecting the best model.

The same results are confirmed with the PEC (Figure 5), where the lower curve is the blue one, which corresponds to the model based on clinical and radiological covariates. It exhibits the lowest mean Brier score (0.097) in comparison to the model based on grading and margin (0.145 with Wilcoxon Rank Sum test *p*-value = 0.002) and to the model estimated on ten radiological features (0.138, with *p*-value = 0.007).

## 4. Discussion

AdCC is a heterogeneous tumor both histologically and clinically, with a complex clinical behavior. AdCC exhibits different histopathological patterns of growth: tubular, cribriform, and solid, with the solid subtype associated with a poorer prognosis. AdCC is often characterized by a slow, relentless growth, often associated with infiltration into surrounding tissues, with a strong propensity to perineural invasion (occurring in 83.7% of cases in our cohort), as well as distant hematogenous spread.

Because of these features, AdCC exhibits a biological behavior that is distant from more common histologies of the head and neck (e.g., squamous cell carcinoma). Its common prolonged asymptomatic clinical course often leads to a delay in diagnosis, with many tumors diagnosed at an advanced stage with extensive perineural outgrowths. Moreover, the risk of local and distant recurrence is high, even years after the primary treatment.

Its peculiar biological behavior makes risk stratification tools developed for other histologies inadequate, while its rarity makes the development of its own prognostic tools difficult. Therefore, the urgent need for a risk stratification tool specific to AdCC was met by identifying a prognostic tool through synthetic data augmentation models using both radiomics and clinical features.

Through the use of radiomics and machine learning, substantial progress has been made in developing predictive models for many malignancies, but not for AdCC. To the best of our knowledge, no studies on the predictive value of radiomics in AdCC have been published in the literature to date. In the field of salivary gland oncology, a few studies have addressed the differential diagnosis between benign and malignant lesions in salivary glands [27,28], but none have specifically considered AdCC.

This study aims to provide a model that combines clinical (tumor grading and margin status) and radiomic features (texture analysis of preoperative MRI) for predicting Relapse-Free Survival (RFS) in AdCC patients.

Considering the survival models developed with our analysis, it is interesting that the model based on clinical covariates (grading and margin, C-index = 0.67) has a similar C-index compared to the models based on radiomics (C-index = 0.68). This result obtained from the radiomic model highlights that radiomics brings significant and independent predictive value compared with traditional clinical factors. Combining the clinical and radiomic models resulted in a significant improvement in the concordance of this model (C-index = 0.77).

This clinical-radiomic model showed a good predictive performance, as illustrated by both the C-index and Brier score in the Prediction Error Curves (PEC) (Table 2 and Figure 5). These results might reflect the complex biological behavior of AdCC, in which both microscopic tumor characteristics (captured by radiomics) and traditional clinical markers (tumor grade and margins) influence outcomes.

In this setting of patients, the integration of radiomics analysis into predictive models could hold significant potential for clinical decision-making in the management of AdCC. Improved prediction of relapse could help physicians better understand the disease and implement better treatment strategies.

In the setting of precision medicine, a predictive model based on a combined clinical-radiomic model can move beyond a “one-size-fits-all” approach to more tailored patient care. The use of radiomics could assist in the earlier identification of patients at risk of metastasis, enabling earlier intervention strategies. In the future, patients identified as high risk based on the combined model could benefit from closer follow-up after surgery, more aggressive adjuvant therapies, or even experimental treatments. On the other hand, low-risk patients could avoid unnecessary treatments, thus reducing the side effects of therapies.

This study has several limitations. Firstly, the relatively small sample size: although this is a common limitation in rare cancers like AdCC, this could reduce the statistical power of the analysis. Although this study provides a robust analysis of Relapse-Free Survival (RFS) using clinical and radiomic predictors for Adenoid Cystic Carcinoma (AdCC) patients, some key confounders were not included in the statistical models due to limitations in the dataset. Despite the fact that synthetic data augmentation techniques helped address this issue, the generalizability of the findings to larger patient populations remains uncertain. Moreover, this study could benefit an external validation cohort. Another limitation was the retrospective nature of the study, which introduces inherent biases related to patient selection and treatment decisions. Prospective studies are needed to validate these findings. Finally, while radiomic features provided added value, the robustness of these features across different imaging protocols and machines must be explored to ensure broader applicability.

## 5. Conclusions

This study is the first to demonstrate the utility of a combined radiomic-clinical model to predict Relapse-Free Survival in AdCC patients.

The results obtained suggest that this model may provide clinicians with a valuable tool in the patient’s management. Accurate risk stratification is of great importance for treatment planning.

## Figures and Tables

**Table 1:** Baseline patient and tumor characteristics.

Patients and Tumor Characteristics		
Gender	Female	63.5%
		Median age 53 (Inter-quartile range 38–61)
Site of origin	Minor salivary glands	79.6%
Subsite of origin	Sinonasal tract	44.9%
	Parotid gland	20.4%
	Oral cavity	18.4%
	Nasopharynx	10.2%
	Other	6.1%
Histologic grading according to Perzin–Szanto	High-grade (G3)	32.7%
	Intermediate-grade (G2)	49.0%
	Low-grade (G1)	18.3%
Tumor staging	pT1	6.1%
	pT2	8.2
	pT3	10.2%
	pT4	75.5%
Local tumor extension	Skin	6.1%
	Named nerves	58.3%
	Muscles	48.9%
	Bones	76.1%
	Cartilage	29.3%
Perineural invasion	Pn1	91.1%
Lymphovascular invasion	Lv1	24.4%
Surgical resection margins	R1	55.6%
	R2	15.6%
N status	N+	18.4%
Extranodal extension	ENE+	14.3%
Median N. of metastastic nodes (IQR)		2 (2–5)
Adjuvant treatment	RT	80.9%
	ChRT	14.9%

**Table 2:** Cox regression estimates for the three estimated models. Clinical variables and the radiomic features included in the three Cox models.

Outcome RFS			
**First Cox model**			
**Covariates**	**HR**	**95% CI**	***p*-value**
**Grading G2**	1.673	(0.624–4.488)	0.306
**Grading G3**	3.722	(1.166–11.884)	***0.026***
**Margin SR1**	2.506	(0.902–6.964)	0.078
**Second Cox model**			
**Covariates**	**HR**	**95% CI**	***p*-value**
**GrayLevelNonUniformity**	1.001	(1.000–1.003)	0.103
**log_sigma_2_0_mm_3DglszmZoneVariance**	1.000	(1.000–1.000)	0.881
**log_sigma_2_0_mm_3DglrlmGrayLevelNonUniformity**	0.996	(0.991–1.000)	0.059
**log_sigma_2_0_mm_3DglszmLargeAreaEmphasis**	1.000	(1.000–1.000)	0.649
**log_sigma_2_0_mm_3DgldmGrayLevelNonUniformity**	1.001	(0.999–1.004)	0.382
**log_sigma_4_0_mm_3DgldmDependenceVariance**	1.034	(0.963–1.111)	0.358
**DependenceEntropy**	2.313	(1.024–5.223)	***0.044***
**log_sigma_4_0_mm_3DfirstorderMaximum**	1.008	(1.002–1.014)	***0.009***
**log_sigma_4_0_mm_3DglrlmRunLengthNonUniformity**	1.000	(0.999–1.000)	0.805
**log_sigma_4_0_mm_3DglrlmRunVariance**	0.949	(0.612–1.471)	0.815
**Third Cox model**			
**Covariates**	**HR**	**95% CI**	***p*-value**
**LevelNonUniformity**	1.000	(0.998–1.002)	0.950
**log_sigma_2_0_mm_3DglszmZoneVariance**	1.000	(1.000–1.000)	0.996
**log_sigma_2_0_mm_3DglrlmGrayLevelNonUniformity**	0.998	(0.992–1.004)	0.495
**log_sigma_2_0_mm_3DglszmLargeAreaEmphasis**	1.000	(1.000–1.000)	0.064
**log_sigma_2_0_mm_3DgldmGrayLevelNonUniformity**	1.002	(0.999–1.005)	0.208
**log_sigma_4_0_mm_3DgldmDependenceVariance**	0.981	(0.902–1.066)	0.651
**DependenceEntropy**	1.633	(0.516–5.168)	0.404
**log_sigma_4_0_mm_3DfirstorderMaximum**	1.013	(1.005–1.022)	***0.002***
**log_sigma_4_0_mm_3DglrlmRunLengthNonUniformity**	0.999	(0.999–1.000)	0.106
**log_sigma_4_0_mm_3DglrlmRunVariance**	0.983	(0.576–1.678)	0.950
**Grading G2**	1.902	(0.518–6.988)	0.333
**Grading G3**	4.154	(0.776–22.228)	0.096
**Margin SR1**	11.634	(2.202–61.454)	***0.004***

In bold and italics, significant *p*-values.

**Table 3:** C-index and standard error (se) for the three Cox models where the outcome is the RFS.

Models	C-Index (se)
Model based on clinical covariates (grading and margin)	0.67 ± 0.07
Model based on radiological features (ten variables selected by SRF)	0.68 ± 0.04
Model based on clinical (grading and margin) and radiological (ten variables selected by SRF) covariates	0.77 ± 0.06

## Data Availability

Data are available from authors on reasonable request.

## Supplementary Materials

The following supporting information can be downloaded at: https://www.mdpi.com/article/10.3390/cancers16233926/s1, Table S1: Univariate Cox analysis for identifying the clinical variables associated with the Relapse-Free Survival (RFS)
