# Supplementary material for: Radiomic and Clinical Model in the Prognostic Evaluation of Adenoid Cystic Carcinoma of the Head and Neck
Source: Cancers (Basel). 2024 Nov 23;16(23):3926. doi: 10.3390/cancers16233926 (PMC11640122; doi:10.3390/cancers16233926)
Supplement: Supplementary file 1 [file cancers-16-03926-s001.zip › cancers-3301178-supplementary.pdf]

Table S1: Univariate Cox analysis for identifying the clinical variables associated with the Relapse-Free Survival (RFS)

Variables	N	HR[1]	95% CI[1]	p-value
GENDER	48			
F		—	—	
M		1.26	0.58, 2.74	0.55
AGE	48	1.01	0.99, 1.03	0.43
SMOKE	43			
EX		—	—	
No		0.99	0.38, 2.53	0.98
Yes		1.07	0.21, 5.33	0.94
ALCOHOL	42			
No		—	—	
Yes		0.40	0.13, 1.18	0.10
BMI	25	0.92	0.80, 1.07	0.30
MAJMIN	48			
MAJ		—	—	
MIN		0.67	0.28, 1.58	0.36
ORIGIN	48			
LARYNX		—	—	
NASOPHARYNX		0.29	0.02, 4.61	0.38
ORAL_CAVITY		0.95	0.11, 7.95	0.96

Variables	N	HR[1]	95% CI[1]	p-value
OROPHARYNX		0.00	0.00, Inf	>0.99
OTHER		0.00	0.00, Inf	>0.99
PAROTID		1.29	0.16, 10.7	0.82
SINONASAL		1.04	0.13, 8.07	0.97
ORIGIN2	47			
MAJOR		—	—	
ORAL_CAVITY		0.73	0.25, 2.19	0.58
PHARYNGOLARYNGEAL		0.32	0.07, 1.54	0.16
SINONASAL		0.81	0.32, 2.03	0.65
LOCALIZATION	48			
HARD_PALATE		—	—	
LARYNX		0.83	0.10, 7.21	0.87
MAXILLARY_SINUS		0.99	0.31, 3.19	>0.99
NASOETHMOIDAL		0.96	0.29, 3.16	0.95
NASOPHARYNX		0.24	0.03, 2.06	0.19
OROPHARYNX		0.00	0.00, Inf	>0.99
OTHER		0.00	0.00, Inf	>0.99
OTHER_ORAL_CAVITY		0.37	0.04, 3.19	0.37
PAROTID		1.06	0.33, 3.37	0.92

Variables	N	HR[1]	95% CI[1]	p-value
SPHENOID_SINUS		0.00	0.00, Inf	>0.99
**ALBUMIN**	37	1.00	0.99, 1.01	0.73
**HB**	40	1.01	0.99, 1.04	0.35
**WBC**	40	1.00	1.00, 1.00	0.96
**NEUTROPH**	39	1.00	1.00, 1.00	0.84
**LYMPHOC**	38	1.00	0.99, 1.01	0.57
**NLR100**	38	1.00	1.00, 1.01	0.66
**PNIndex10**	36	1.00	0.99, 1.01	0.45
**Albumin2**	37	1.24	0.49, 3.12	0.65
**Hb2**	40	1.12	0.88, 1.43	0.35
**NLR2**	38	1.10	0.72, 1.68	0.66
**PNIndex2**	36	1.04	0.94, 1.14	0.45
**SUBTYPE**	48			
CRIBRIFORM		—	—	
MIXED		0.61	0.13, 2.91	0.54
SOLID		1.58	0.64, 3.91	0.32
TUBULAR		0.38	0.05, 3.02	0.36

Variables	N	HR[1]	95% CI[1]	p-value
**TUBULAR_CRIBRIFORM**		0.45	0.15, 1.39	0.17
**GRADING**	48			
**G1**		—	—	
**G2**		2.39	0.75, 7.56	0.14
**G3**		4.43	1.39, 14.0	***0.012***
**MULTINODULAR**	39			
**No**		—	—	
**Yes**		0.80	0.33, 1.94	0.62
**SATELLITOSIS**	36			
**No**		—	—	
**Yes**		0.87	0.29, 2.66	0.81
**pT**	48			
**T1**		—	—	
**T2**		56,156,409	0.00, Inf	>0.99
**T3**		25,639,752	0.00, Inf	>0.99
**T4a**		78,317,621	0.00, Inf	>0.99
**T4b**		107,277,229	0.00, Inf	>0.99
**pT4b**	48	1.82	0.86, 3.85	0.12
**pN**	48			
**cN0_pN0**		—	—	
**N2**		3.23	0.71, 14.6	0.13

Variables	N	HR[1]	95% CI[1]	p-value
N3		1.87	0.64, 5.46	0.25
STAGING	48			
I		—	—	
II		56,156,409	0.00, Inf	>0.99
III		25,639,752	0.00, Inf	>0.99
IVa		78,317,621	0.00, Inf	>0.99
IVb		107,277,229	0.00, Inf	>0.99
STAGEIVb	48			
0		—	—	
IVb		1.82	0.86, 3.85	0.12
Bilateral_Extension	47			
No		—	—	
Yes		0.90	0.31, 2.66	0.85
Peri_glandular_soft_tissues	41			
No		—	—	
Yes		0.84	0.36, 1.97	0.70
SKIN	48			
No		—	—	
Yes		1.30	0.31, 5.51	0.72
PNI	44			
No		—	—	
Yes		78,154,761	0.00, Inf	>0.99

Variables	N	HR[1]	95% CI[1]	p-value
**PNI2**	41			
**0**		—	—	
**A**		91,819,918	0.00, Inf	>0.99
**B**		62,491,190	0.00, Inf	>0.99
**PNI3**	44			
**0**		—	—	
**1**		49,520,655	0.00, Inf	>0.99
**B**		111,898,140	0.00, Inf	>0.99
**LVI**	40			
**No**		—	—	
**Yes**		1.95	0.76, 5.04	0.17
**BCINVASION**	43			
**0**		—	—	
**B_Ci**		2.82	0.96, 8.31	0.060
**SubPP_Ci**		0.93	0.31, 2.77	0.89
**NECROSIS**	44			
**No**		—	—	
**Yes**		1.90	0.75, 4.80	0.17
**MARGINS**	**44**			
**R0**		—	—	
**R1**		4.12	1.39, 12.2	***0.011***
**NPOS**	48			

Variables	N	HR[1]	95% CI[1]	p-value
No		—	—	
Yes		2.24	0.94, 5.34	0.068
TOTMAJDIAM	9	1.05	0.95, 1.16	0.37
TOTENE	48			
0		—	—	
ENE_Minus		2.00	0.26, 15.5	0.50
ENE_plus		2.29	0.92, 5.71	0.077
LC_N_plus	48			
No		—	—	
Yes		2.24	0.94, 5.34	0.068
ENE_plus	48			
0		—	—	
ENE_minus		2.00	0.26, 15.5	0.50
ENE_plus		2.29	0.92, 5.71	0.077
Parotid_N_plus	48			
No		—	—	
Yes		3.87	0.89, 16.9	0.072
Numb_of_removed_N2	46	1.00	0.89, 1.13	0.97
ENE_plus2	43			
0		—	—	

Variables	N	HR[1]	95% CI[1]	p-value
ENE_plus		3.84	0.87, 16.9	0.076
ADJTREATMENT	46			
0		—	—	
CHRT		142,472,325	0.00, Inf	>0.99
RT		72,868,866	0.00, Inf	>0.99
ADJRT	46			
No		—	—	
Yes		76,184,703	0.00, Inf	>0.99
ADJCHT	39			
No		—	—	
Yes		2.24	0.81, 6.19	0.12
REGIONAL_REC	44			
No		—	—	
Yes		2.86	0.84, 9.74	0.093
[1] HR = Hazard Ratio, CI = Confidence Interval				

In bold and Italics p-values <0.05
